# The relationship between college students’ psychological resilience and autonomous fitness behavior: a moderated mediation model

**DOI:** 10.3389/fpsyg.2025.1513031

**Published:** 2025-06-04

**Authors:** Xiaoheng Wang, Xiang Zhao, Na Li

**Affiliations:** ^1^School of Physical Education, Chizhou University, Chizhou, China; ^2^School of Physical Education, Huaibei Normal University, Huaibei, China; ^3^School of Physical Education, Shandong University of Science and Technology, Qingdao, China

**Keywords:** psychological resilience, perceived social support, autonomous fitness behavior, exercise self-efficacy, college students

## Abstract

**Objective:**

This paper aims to explore the influence of psychological resilience on autonomous fitness behavior among college students, as well as the mechanism of perceived social support and exercise self-efficacy in this relationship.

**Methods:**

Using literature review, questionnaire surveys, and mathematical statistics, we conducted a survey among 985 college students, administering the Psychological Resilience Scale, Autonomous Fitness Behavior Scale, the Perceived Social Support Scale, and the Exercise Self-efficacy Scale.

**Results:**

(1) College students’ psychological resilience has a direct impact on their autonomous fitness behavior and can positively predict it (*β* = 0.833, *t* = 14.680, *p* < 0.001); (2) Perceived social support plays a partial mediating role between psychological resilience and autonomous fitness behavior among college students, with a mediating effect value of 0.288 (*t* = 21.415, *p* < 0.001); (3) Exercise self-efficacy regulates the first half of the mediating path of “psychological resilience → perceived social support → autonomous fitness behavior” (*β* = 0.545, *t* = 14.680, *p* < 0.001). The interaction between psychological resilience and exercise self-efficacy affects perceived social support, which in turn indirectly affects autonomous fitness behavior. Under the regulation of this mediating model, the predictive effect of psychological resilience on autonomous fitness behavior varies significantly across different levels of self-efficacy.

**Conclusion:**

(1) Psychological resilience can directly promote college students’ autonomous fitness behavior, and can promote their autonomous fitness behavior through perceived social support. Perceived social support plays a partial mediating role between psychological resilience and autonomous fitness behavior, and this mediating effect can be moderated by exercise self-efficacy. (2) Compared with high exercise self-efficacy, for college students with low exercise self-efficacy, perceived social support plays a stronger mediating role.

## Introduction

In order to achieve the era goal of “holistic health,” the ‘Healthy China 2030’ Planning Outline emphasizes the comprehensive life-cycle maintenance and guarantee of people’s health. College students’ physical fitness is not only related to the construction of a sports powerhouse, but also a significant symbol of the country’s comprehensive strength, which directly affects the country’s future and national development. Although the state has launched a series of policies and measures, the physical fitness of adolescents has not seen significant improvement. They lack fundamental sports skills, such as fast running, swimming, and three-step slam dunk. They cannot juggle a ball, climb trees, or even find it difficult to complete a standard pull-up. In this context, autonomous fitness behavior, as a consciously chosen, optimized, and compensated individual fitness goal and means, is particularly critical. It is an important way to solve the health problems of adolescents. The development of autonomous fitness behavior is influenced by many internal and external factors. Intrinsic factors include self-motivation and psychological resilience, while extrinsic factors include the encouragement and support of significant others such as family and friends, as well as individuals’ subjective feelings of understanding, respect, and support. These factors subtly shape college students’ cognition and values, and then affect the occurrence of autonomous fitness behavior.

### Relationship between psychological resilience and autonomous fitness behavior

Psychological resilience refers to the remarkable adaptability exhibited by individuals when faced with pressures, difficulties, threats, and other adverse situations. It serves as a positive factor in safeguarding mental health and a crucial force in actively tackling life’s challenges. As a focal point of research in the field of positive psychology, the exploration of psychological resilience has garnered widespread attention from international scholars. For instance, Olsson et al. (2003) posited in his study that psychological resilience is not isolated. It encompasses multiple influencing factors such as personal ability and personality traits, family support system, social support system, etc., which together constitute the rich connotation and multiple structure of psychological resilience. During the growth process of college students, they are bound to encounter numerous challenges and difficulties. Individuals with high psychological resilience are capable of finding motivation in negative events or misfortunes, thereby transforming adverse factors into catalysts for growth. This ability allows them to transform adversity into a source of strength (Weber et al., 2020). In sports competitions, psychological resilience also plays a pivotal role. Athletes with high psychological resilience are adept at adjusting emotional information and demonstrating exceptional emergency handling skills (Chen and Wang, 2014). Research indicates that a high level of psychological resilience is one of the key characteristics of successful athletes (Wu et al., 2024). During competitions, they need to efficiently manage pressure, and strong psychological resilience can alleviate their perception of pressure, enabling them to adopt more effective coping strategies. Therefore, psychological resilience is not only the key psychological quality for athletes to achieve excellent results, but also an important guarantee for them to mobilize all kinds of resources and respond flexibly to different pressures in the face of difficulties and setbacks. Based on the above discussion, we can see that the positive benefits brought by psychological resilience can not only help us cope with the pressures and challenges in sports, but also help us form stable emotions and good adaptability, better regulate stress behavior, and participate in sports activities more actively. Accordingly, Hypothesis 1 proposes: psychological resilience can directly affect college students’ autonomous fitness behavior and become a powerful driving force for them to actively participate in sports.

### The mediation effect of perceived social support

Social support, which includes the objective and subjective assistance that individuals receive from all aspects of society, is a key indicator to measure the degree and quality of personal and social ties (Chen et al., 2016). The individual’s expectation and perception of receiving support from significant others is called perceived social support. It affects individual emotions and behaviors through positive, stable, and lasting forces, and plays an important role in promoting emotional engagement in college students’ sports activities (Wang et al., 2019). Previous studies have confirmed that family support is a positive indicator of adolescent sports activities (Draper et al., 2015). Adolescents’ perceived support from parents and friends can promote their regular participation in sports activities (Wen et al., 2024). In sports training, athletes’ perceived support from coaches is positively correlated with their long-term exercise adherence (Pelletier et al., 2001). While students’ perception of physical education teacher support can positively predict their participation in extracurricular sports activities (Hagger et al., 2013). Peer support, including classmates and friends, is also positively correlated with sports participation (Bronikowski et al., 2016). The absence of such support poses as a significant barrier to college students’ sports participation (Abdelghaffar et al., 2019). Hence, perceived social support is considered a direct factor in promoting physical fitness among college students. Psychological resilience, as a trait and ability to cope with adverse life events, enables college students with high resilience to demonstrate a higher level of cognition and emotional control when adjusting emotional information and facing setbacks. They possess a deeper comprehension of family and social support. This understanding and perceived support offer protection, bolster positive psychological experiences, and facilitate the maintenance and development of autonomous fitness behavior. Enhancing college students’ psychological resilience can deepen their understanding of social support, thereby promoting the development of autonomous fitness behavior. Therefore, hypothesis 2 is proposed: Perceived social support plays a mediating role between college students’ psychological resilience and autonomous fitness behavior.

### The moderation effect of exercise self-efficacy

Exercise self-efficacy, as a core component of social cognitive theory, refers to the beliefs and perceptions individuals hold regarding the achievement of their expected behavioral goals (Bandura, 1977). This concept serves not only as a key determinant in the formation of motivation but also as a core driving force in behavioral activities. It can regulate individuals’ cognition, attitudes, and emotions, and influence their behavioral intentions through the provision of motivation (Qu, 2021). The level of exercise self-efficacy determines an individual’s perception and response to social support, subsequently influencing their implementation of autonomous fitness behavior (Li et al., 2024). Specifically, college students with high self-efficacy are generally more confident and believe that they have the ability to improve their physique and health through fitness activities. This self-confidence encourages them to actively seek and use social support resources, such as the support and encouragement of family, friends, and campus associations, so as to participate in self-directed fitness more effectively. At the same time, the high level of exercise self-efficacy also improves the individual’s ability to cope with the challenges of fitness, so that they can maintain a positive attitude and continue to work hard in the face of difficulties and setbacks.

Research showed that athletes with strong psychological resilience tend to use psychological adjustment strategies in the face of pressure, such as relaxation techniques and self-suggestion, to improve their psychological state by adjusting their cognition and attitude, so as to improve their sense of exercise self-efficacy and further adjust their ability to understand social support (Xie and Yang, 2021). Therefore, exercise self-efficacy plays a moderating role between psychological resilience and perceived social support, affecting the strength and direction of the relationship between them. Specifically, a high level of exercise self-efficacy can enhance the positive impact of psychological resilience on perceived social support. When individuals have a high sense of exercise self-efficacy, they are more likely to turn external support and encouragement into behavioral motivation, so as to participate in fitness activities more actively. In addition, exercise self-efficacy may also regulate the relationship between psychological resilience and perceived social support, and affect individuals’ commitment to fitness behavior. Research has found that exercise self-efficacy can positively predict fitness activity adherence, meaning that the higher the self-efficacy, the better the fitness activity adherence (Tang and Guo, 2021). This indicates that exercise self-efficacy has an amplifying effect in the process of psychological resilience and understanding the impact of social support on fitness behavior. The moderating effect of self-efficacy is also evident in its ability to influence individuals’ cognitive and emotional responses to fitness behavior. Individuals with high exercise self-efficacy are more likely to experience positive emotions and satisfaction in the face of fitness challenges, which in turn enhances their psychological resilience and the ability to understand social support. Therefore, this study proposes hypothesis 3: self-efficacy moderates the relationship between college students’ psychological resilience and perceived social support. This hypothesis is proposed to further investigate the mechanism by which self-efficacy influences fitness behavior through psychological resilience and perceived social support, thereby providing a theoretical foundation for promoting autonomous fitness behavior among college students.

**Figure 1:**
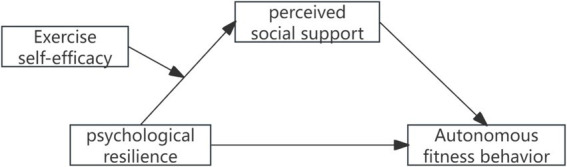
Conceptual model.

In summary, this study aims to establish a moderated mediation model to explore the mediating (perceived social support) and moderating (exercise self-efficacy) mechanisms of psychological resilience on college students’ autonomous fitness behavior, thereby providing theoretical guidance for its development and enhancement (Figure 1).

## Materials and methods

### Participants and procedure

This study employed an online questionnaire survey to investigate adolescents from eight provinces, namely Beijing, Shandong, Anhui, Liaoning, Henan, Jiangsu, Zhejiang, and Guangdong. The sampling was conducted using a convenient random sampling method, where participants were recruited through various online platforms and university networks to ensure a diverse geographical representation. Participants were selected based on the following criteria: (1) being aged 17–22 years, (2) currently enrolled as freshmen in higher education institutions, and (3) residing in the target provinces during the survey period. These criteria ensured the representativeness of the regional adolescent population. After excluding invalid questionnaires (e.g., those with regular responses), a total of 985 valid questionnaires were obtained. The gender distribution of the participants was as follows: 298 were males (30.3%), and 687 were females (69.7%). The average age was 19.55 years. In terms of geographical origin, 237 adolescents (24.1%) came from provincial and prefecture-level cities, 355 (36.0%) from county-level cities, and 393 (39.9%) from rural towns and villages.

The entire surveying process was approved by school leaders, class teachers, and the participants themselves. The study strictly adhered to the principles of voluntary participation, data confidentiality, and anonymity, and was conducted through a collective testing approach. Prior to this, the study had been approved by the Ethical Committee of the Shandong University of Science and Technology, and the questionnaire included control variables such as participants’ age, gender, and geographical origin. It took each participant approximately 5–10 min to fill out the questionnaire, and all invitees participated voluntarily.

### Measures

#### Mental toughness scale

This study also uses the Adolescent Resilience Scale compiled by Yueqin and Yiqun (2008), which contains 27 items and adopts a Likert 5-level scoring method, with scores ranging from 1 (strongly disagree) to 5 (strongly agree). The scale covers five dimensions: goal focus, interpersonal assistance, family support, emotional regulation, and positive cognition. According to the scale, the internal consistency reliability *α* is 0.83. After statistical analysis, the internal consistency reliability α of the scale is 0.938, indicating that the scale has good reliability. In this study, the scale demonstrated robust internal consistency, with an overall Cronbach’s α coefficient of 0.843. Confirmatory factor analysis results further supported the scale’s validity, with model fit indices as follows: CMIN/DF = 2.984, GFI = 0.925, CFI = 0.961, TLI = 0.957, and RMSEA = 0.045. These findings collectively provide strong evidence for the scale’s reliability and validity, confirming its suitability for assessing psychological resilience among university students.

#### Autonomous fitness behavior scale

In this study, the Autonomous Fitness Behavior developed by Fang et al. (2012) was used. The scale includes three dimensions: self-determination (*α* = 0.859), autonomy support (α = 0.786), and self-regulation SOC strategies (α = 0.876), with a total of 43 items. The scale adopts a 5-level scoring method, with scores ranging from 1 (strongly disagree) to 5 (strongly agree). The scale items included the following statements: (1) “Fitness is a hobby that I genuinely enjoy,” (2) “I possess comprehensive knowledge and practical skills related to fitness,” and (3) “During physical activities, I demonstrate sustained energy levels and strong physical stamina.” In this study, the questionnaire exhibited strong internal consistency reliability, with a Cronbach’s alpha of 0.783. Further analysis revealed high internal consistency across the subdimensions: sense of self-determination (*α* = 0.943), sense of autonomy support (α = 0.931), and self-regulation through SOC strategies (α = 0.954). Confirmatory factor analysis results indicated a good model fit, with the following indices: CMIN/DF = 2.021, GFI = 0.912, CFI = 0.967, TLI = 0.965, and RMSEA = 0.032. These results suggest that the scale is highly reliable and valid for assessing different dimensions of autonomous fitness behavior among university students, thereby meeting the requirements for psychometric rigor.

#### Perceived social support scale

The social support multidimensional scale MSPSS used in this study was compiled by Zimet et al. (1987) and later translated and revised by and Qianjin (1999). It is a tool for measuring the degree of social support perceived by individuals from multiple aspects, comprising three dimensions and twelve items. The scale adopts a 7-point rating scale, where 1 represents “completely non-conforming” and 7 represents “fully conforming.” The scale items encompass the following statements: (1) “When I encounter difficulties, there are people (such as teachers, classmates, or relatives) who stand by me,” (2) “I can share both joys and sorrows with certain individuals (including teachers, classmates, or relatives),” and (3) “My family is able to provide me with concrete support when needed.” Higher total scores indicate higher levels of perceived social support. The scale demonstrated strong internal consistency, with a Cronbach’s alpha coefficient of 0.930. The internal consistency reliabilities of the subscales for family support, friend support, and other support were 0.830, 0.820, and 0.760, respectively. Confirmatory factor analysis results showed excellent model fit, with the following indices: CMIN/DF = 2.135, GFI = 0.932, CFI = 0.901, TLI = 0.902, and RMSEA = 0.034. These findings provide compelling evidence of the scale’s reliability and validity, confirming its appropriateness for assessing perceived social support across multiple dimensions.

#### The exercise self-efficacy scale

Kroll et al. (2007) developed the Exercise Self-Efficacy Scale (ESES), which was subsequently revised by Yanjin et al. (2016). The scale includes 10 items and adopts a Likert 4-level scoring method, where 1 indicates “completely non-conforming” and 4 indicates “completely conforming.” The scale items include the following statements: (1) “If I try my best, I can always solve the problems that arise during exercise,” (2) “I am confident in finding an exercise method that suits me,” and (3) “I am confident in achieving the exercise goals I set for myself each day.” The internal consistency coefficient *α* of the scale was 0.879, the split-half reliability coefficient r was 0.858, and the test–retest reliability was 0.657. In this study, the internal consistency coefficient α of the scale was 0.948. Confirmatory factor analysis yielded satisfactory model fit indices, with CMIN/DF = 2.221, GFI = 0.901, CFI = 0.927, TLI = 0.920, and RMSEA = 0.027. These results provide robust evidence of the reliability and validity of the ESES.

### Statistical analyses

(1) Descriptive and Correlational Analysis: IBM SPSS26.0 statistical software was used for data analysis, including descriptive statistics and correlation analysis of perceived social support, autonomous fitness behavior, psychological resilience, exercise self-efficacy and other variables; To ensure the reliability of our findings, we employed Harman’s single-factor test to assess common method bias.

(2) Mediation and Moderation Analysis with PROCESS: Model 4 and Model 7 in the macro program PROCESS of SPSS was used to conduct the mediating effect test. The primary tests include: the direct effect of psychological resilience on autonomous fitness behavior; the mediating role of perceived social support in this relationship; the moderating effect of exercise self-efficacy on the relationship between psychological resilience and perceived social support.

(3) Structural Equation Modeling with AMOS: AMOS offered a more comprehensive assessment of the overall model fit and relationships among the variables.

## Results

### Common method deviation test

This study was conducted using a questionnaire survey. To detect and control potential common method biases, we included 12 reverse items in the “Psychological Resilience Scale” and 9 reverse items in the “Autonomous Fitness Behavior Scale” during the design of the questionnaire. During data collection, we ensured data accuracy through various methods such as on-site filling out, on-site answering questions, and on-site questionnaire collection. Additionally, to further verify the issue of common method biases, this study employed the Harman’s single-factor test method for data analysis. By extracting all items into a single-factor unrotated exploratory factor analysis, we found that there were 10 factors with eigenvalues greater than 1, with the largest factor explaining 39.31% of the variance. This proportion is lower than the 40% criterion proposed by Hair et al., indicating that there is no significant common method bias in this study.

### Descriptive statistical and correlation analysis

Table 1 presents the mean, standard deviation, and Pearson product–moment correlation matrix for each variable. The analysis results indicate that there is a significant positive correlation between psychological resilience, exercise self-efficacy, perceived social support, and autonomous fitness behavior (*p* < 0.01). Additionally, there is a significant positive correlation between gender and age, psychological resilience, exercise self-efficacy, and autonomous fitness behavior (*p* < 0.01), while age shows a significant negative correlation with psychological resilience (*p* < 0.01). Further gender-stratified analyses revealed distinct patterns: male students reported higher baseline levels of exercise self-efficacy, psychological resilience and autonomous fitness frequency. Given these observed disparities, gender and age were subsequently included as covariates in multivariate analyses to account for potential confounding effects.

**Table 1:** Descriptive statistics and correlation analysis (*n* = 985).

Relevant variable	1	2	3	4	5	6
1. Gender	1.000					
2. Age	0.094^**^	1.000				
3. Psychological resilience	0.145^**^	−0.057^*^	1.000			
4. Exercise self-efficacy	0.197^**^	−0.019	0.572^**^	1.000		
5. Perceived social support	−0.038	−0.033	0.596^**^	0.542^**^	1.000	
6. Autonomous fitness behavior	0.185^**^	−0.032	0.657^**^	0.826^**^	0.647^**^	1.000
*M*	1.460	19.600	3.6827	2.931	5.464	3.580
SD	0.499	1.665	0.586	0.620	1.105	0.786

**p < 0.01, *p < 0.01, gender: 1 = female, 2 = male.

### Mediation effect test

**Figure 2:**
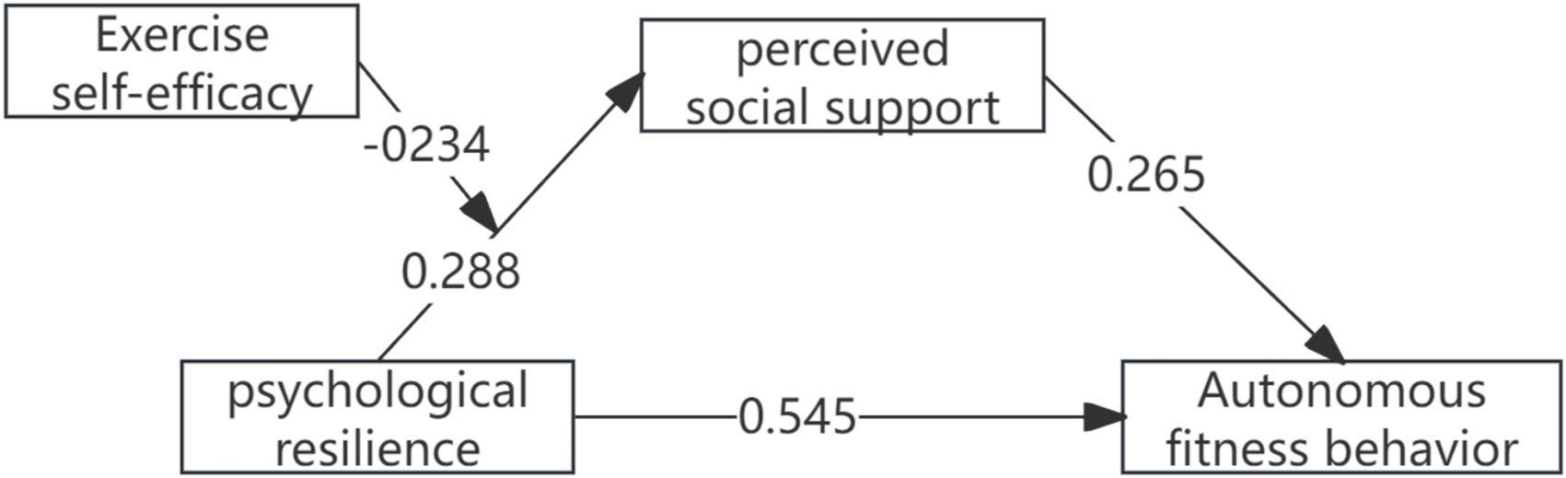
Moderated mediation model.

This study adopted Model 4 from the SPSS macro program PROCESS v3.5, developed by Hayes (2013). After controlling for the effects of age and gender, a mediation effect test was conducted, using psychological resilience as the independent variable, autonomous fitness behavior as the dependent variable, and perceived social support as the mediating variable. The results, as presented in Table 2, indicate that psychological resilience significantly and positively impacts autonomous fitness behavior (*β* = 0.833, *t* = 14.680, *p* < 0.001) and perceived social support, while also demonstrating comparable influence on perceived social support (*β* = 0.288, *t* = 21.415, *p* < 0.001). When both psychological resilience and perceived social support are included in the regression equation, the positive predictive effect of perceived social support on autonomous fitness behavior is significant (*β* = 0.265, *t* = 13.756, *p* < 0.001), while the direct predictive effect of psychological resilience on autonomous fitness behavior remains significant (*β* = 0.545, *t* = 14.680, *p* < 0.001). The mediation effect analysis reveals that the mediation effect of perceived social support between psychological resilience and autonomous fitness behavior is 0.288, with a 95% confidence interval of [0.227, 0.350], indicating that the mediation effect accounts for 34.5% of the total effect (Table 3; Figure 2). All reported coefficients are standardized.

**Table 2:** Test of the mediation effect of social support (*n* = 985).

Relevant variable	Equation 1 (Autonomous fitness behavior)		Equation 2 (Perceived social support)		Equation 3 (Autonomous fitness behavior)	
	*β*	*t*	*β*	*t*	*β*	*t*
Gender	0.149	3.687***	−0.270	0.061***	0.221	5.899***
Age	0.004	0.315	0.006	0.362	0.002	0.185
Psychological resilience	0.833	14.680***	0.288	21.415***	0.545	14.680***
Perceived social support	–	–	–	–	0.265	13.756***
*R* ^2^	0.408		0.320		0.503	
*F*	224.927***		153.810***		248.366***	

***p < 0.001.

**Table 3:** Total effect, indirect effect and direct effect.

Action effect	Effect	SE	*p*	LLCI	ULCI
Total effect	0.834	0.034	0.000	0.768	0.899
Direct effect	0.545	0.037	0.000	0.472	0.618
Indirect effect	0.288	0.031	0.000	0.227	0.350

### Moderation effect test

Based on Wen Zhonglin’s data testing requirements and theoretical hypotheses (Lieber et al., 2024), this study adopted Model 7 from the SPSS macro program PROCESS v3.5 developed by Hayes (2013). By drawing 5,000 samples to estimate the Bootstrap 95% confidence interval, we standardized all predictive variables to avoid multicollinearity, and controlled for factors such as age and gender. The results are shown in Table 4. The results of Equation 1 are significant (*F* = 121.930, *p* < 0.001), indicating that psychological resilience (*β* = 0.874, *t* = 14.801, *p* < 0.001) and self-efficacy (*β* = 0.491, *t* = 9.245, *p* < 0.001) have a significant predictive effect on perceived social support, and the interaction term between psychological resilience and self-efficacy also has a significant predictive effect on perceived social support (*β* = −0.234, *t* = −3.711, *p* < 0.001). The results of Equation 2 are also significant (*F* = 248.366, *p* < 0.001), showing that psychological resilience (*β* = 0.545, *t* = 14.680, *p* < 0.001) and perceived social support (*β* = 0.265, *t* = 13.756, *p* < 0.001) have a significant predictive effect on autonomous fitness behavior. This indicates that exercise self-efficacy plays a moderating role between psychological resilience and perceived social support.

**Table 4:** Regulated mediation effect test (*n* = 985).

Relevant variable	Equation 1 (Perceived social support)		Equation 2 (Autonomous fitness behavior)	
	*β*	*t*	*β*	*t*
Gender	−0.303	−5.049***	0.221	5.899***
Age	0.003	0.155	0.002	0.185
Psychological resilience	0.874	14.801***	0.545	14.680***
Exercise self-efficacy	0.491	9.245***	–	–
Psychological resilience*Exercise self-efficacy	−0.234	−3.711***	–	–
Perceived social support	–	–	0.265	13.756***
*R* ^2^	0.384		0.503	
*F*	121.930***		248.366***	

***p < 0.001.

**Figure 3:**
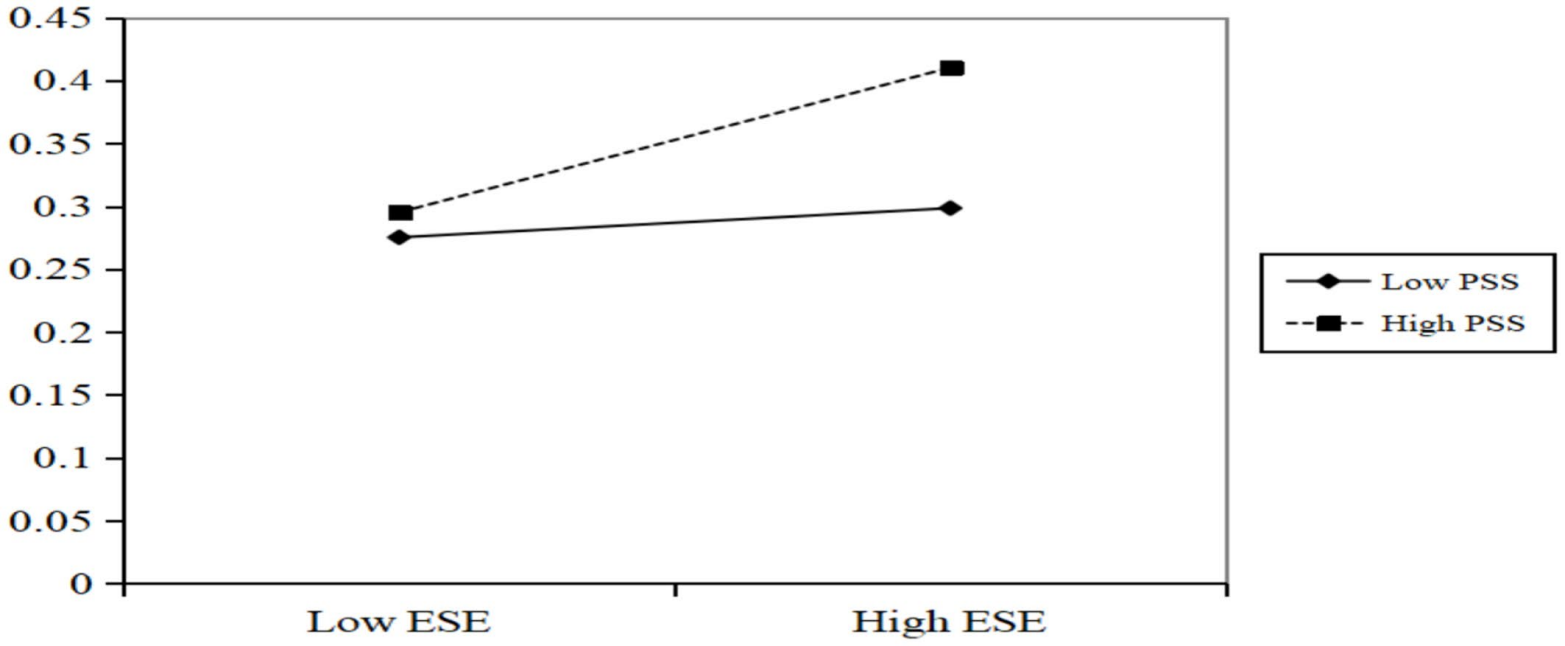
The regulation of exercise self-efficacy on the relationship between psychological resilience and perceived social support.

To further explore the regulatory effect of exercise self-efficacy, exercise self-efficacy was divided into high group (M + 1SD) and low group (M-1SD) according to the principle of adding or subtracting one standard deviation from the average (M ± SD), and a simple slope test was conducted (see Figure 3). The results showed that for college students with low self-efficacy, Psychological resilience had a significant positive predictive effect on perceived social support (*β* = 0.268, SE = 0.037, *p* < 0.001). For students with high self-efficacy, the positive predictive effect was also significant (*β* = 0.195, SE = 0.017, *p* < 0.001), but the prediction coefficient decreased. This indicates that as college students’ exercise self-efficacy increases, the predictive effect of perceived social support on positive coping styles gradually weakens.

Finally, the conditional indirect effects under different exercise self-efficacy levels were calculated. The results showed that when the level of exercise self-efficacy was low, the mediation effect of perceived social support was 0.262 [SE = 0.038, 95% CI (0.187, 0.335)]. However, when the level of exercise self-efficacy was high, the mediation effect was 0.175 [SE = 0.016, 95%CI (0.037, 0.101)]. In addition, the difference between the mediation effect in the two conditions was-0.087 [SE = 0.031, 95%CI (−0.147, −0.026)], and this difference was significant. Thus, the mediation effect of understanding social support increases significantly with the decrease of exercise self-efficacy.

In summary, the moderated mediation model constructed by the study is valid. Among them, perceived social support plays a partial mediating role between psychological resilience and college students’ autonomous fitness behaviors, while exercise self-efficacy moderates the relationship between psychological resilience and perceived social support.

## Discussion

The findings of this study provide robust empirical support for all proposed hypotheses. Specifically, the results indicate that psychological resilience significantly influences college students’ autonomous fitness behaviors both directly and indirectly via perceived social support, with exercise self-efficacy acting as a moderating factor in this relationship. These findings underscore the pivotal roles of psychological resilience, social support systems, and exercise self-efficacy in shaping the development of healthy behaviors among college students.

### The relationship between psychological resilience and autonomous fitness behavior

The study found that psychological resilience can significantly and positively predict college students’ autonomous fitness behavior, which verifies hypothesis 1 and is consistent with the research of other scholars (Xiang, 2013; Na et al., 2023). Research posits that psychological resilience entails the disruption and subsequent reconstruction of an individual’s psychological equilibrium following exposure to stressors. Such stressors may incite dysfunctional cognitive responses, unsettling the individual’s initial psychological balance and compelling them to activate inherent protective resources (Zhou et al., 2024). This activation facilitates the reformation of cognitive and belief systems, culminating in the establishment of a novel equilibrium. Robust psychological resilience not only aids in the maintenance or restoration of psychological and physiological homeostasis amidst adversity but also expedites recovery from setbacks and fosters personal growth. In the realm of sports psychology, mental toughness is characterized as an athlete’s capacity to sustain self-assurance, focus, and judgment amidst stressful conditions. This psychological attribute is efficacious in both adverse and favorable scenarios, contributing to the mitigation of athletic fatigue, the stimulation of enthusiasm for sports, and the adoption of a more positively inclined and optimistic stance towards training and competition (Ma and Mao, 2017).

Within the domain of fitness behavior, psychological resilience signifies an individual’s adaptive and responsive capabilities to challenges across diverse environmental contexts, including family, school, and community. Individuals possessing heightened psychological resilience are prone to leveraging surrounding resources to bolster their fitness pursuits and demonstrate superior adaptability and resilience when confronted with obstacles. This enhances their propensity to adhere to fitness regimens, even in the face of difficulties, reducing the likelihood of abandonment. The phenomenon can be elucidated through the lens of emotional regulation: individuals with elevated psychological resilience are inclined to employ strategies such as cognitive reappraisal to modulate emotions during fitness activities. This modulation sustains a positive emotional state and fortifies the intrinsic motivation and persistence underlying fitness behavior. Furthermore, these individuals exhibit a heightened goal orientation when setting and pursuing explicit fitness objectives, amplifying the autonomy and durability of their fitness practices. They are also more susceptible to experiencing affirmative psychological sensations during fitness activities, such as a sense of accomplishment and contentment, which augment their affinity for fitness activities and bolster voluntary engagement in fitness behavior. Ultimately, individuals with robust psychological resilience are more predisposed to seek out and capitalize on social support, including encouragement and assistance from family members, friends, and coaches. This support furnishes additional motivation and resources for their fitness endeavors.

### The mediation effect of perceived social support

The results of this study show that perceived social support plays a mediating role between psychological resilience and adolescents’ autonomous fitness behavior, which is consistent with hypothesis 2. The study found that perceived social support is one of the key variables affecting young people’s sports activities. Appropriate social support is an effective way to promote young people’s participation in sports activities (Yan and Xu, 2024), and social support is a significant positive indicator to predict Chinese students’ physical activities (Ren et al., 2020). Perceived support from parents, peers, and teachers is significantly associated with physical activity (De Oliveira Barbosa et al., 2024). Perceived social support refers to the support system that college students perceive as meeting their basic fitness needs. This support is not only accepted and understood by college students, but also profoundly affects their basic psychological needs, thereby stimulating their enthusiasm for active participation and promoting their awareness of continuous, stable, and positive engagement in sports activities. This study found that adolescents’ autonomous promotion of sports fitness behavior is significant, which is closely related to the importance of perceived social support (Miao et al., 2022). This conclusion coincides with the results of scholars in the field of training, further verifying the key role of social support in college students’ sports participation. According to the theory of pressure buffering, when individuals perceive social support, they will make a benign assessment of unknown events, so as to effectively deal with potential negative emotions in the process of behavior (Lin and Yeh, 2014). Social support plays a protective role, providing positive coping attitudes and encouragement to enhance individuals’ self-confidence and cognitive evaluation. This kind of positive feedback not only improves the adaptability of individuals, but also reduces the negative experience in the process of behavior (Lerman and Kern, 1983). In addition, drawing on the theories of planned behavior and hedonic theory, the social support perceived by adolescents can shape positive emotional attitudes, and this change in emotional cognition will affect their behavioral beliefs. Due to the high susceptibility of adolescent groups, their emotional attitudes are vulnerable to the influence of mainstream social culture, resulting in a sense of belonging, which has become a key factor in stimulating motivation and guiding behavioral outcomes (Weiner, 1986), and underpins the direction of behavioral intention. Based on a deep understanding of the characteristics and significance of sports activities, college students have formed a positive attitude towards physical fitness and sports behavior, thereby stimulating the emergence of autonomous fitness behavior (Chen et al., 2023).

Psychological resilience, as a protective mechanism against stress responses, is an inherent trait of individuals. It is usually closely related to a variety of protective factors, including both internal ones (such as optimism, self-efficacy, flexible coping strategies) and external ones (such as social support, positive environmental resources). The dynamic model of psychological resilience emphasizes that when facing life challenges, these protective factors can play a timely role in reducing or adjusting adverse effects. The external protective resources provided by society and family for individuals, such as a sense of security, emotional support, and a sense of belonging, not only help to cultivate college students’ abilities but also promote their physical and mental health and help them better adapt to the environment. The internal factors such as self-esteem and emotion regulation, as important supports for psychological resilience, have a positive impact on individual psychological resilience (Yongb et al., 2021). In the growth process of college students, internal and external protective factors complement each other. When external support is perceived by individuals, their self-esteem, emotions, and other internal protective factors are enhanced, thereby making individuals more internally resilient, less susceptible to negative factors, and exhibiting good psychological adaptability. With the enhancement of psychological resilience, college students will have stronger adjustment abilities and understanding, and are better able to perceive and grasp the social support provided by the external environment.

### The moderating effect of exercise self-efficacy

The purpose of this study was to test the mediating effect of perceived social support and to explore the moderating effect of exercise self-efficacy on this mediating process. The results show that exercise self-efficacy plays a moderating role in the first half of the mediating process, indicating that college students with high exercise self-efficacy can enhance their psychological resilience and more effectively perceive social support, thereby promoting autonomous fitness behavior. Exercise self-efficacy refers to the level of confidence individuals have in completing a specific task. During physical exercise, exercise self-efficacy can improve an individual’s commitment to following a fitness plan, making it difficult to give up even in the face of challenges, thereby continuously enhancing psychological resilience. Individuals with high exercise self-efficacy tend to use cognitive reappraisal strategies to regulate emotions, maintain a positive attitude, and enhance the internal motivation and sustainability of fitness. In addition, family support and the support of significant others are key factors in maintaining exercise behavior. Due to the interaction between perceived social support and exercise self-efficacy, exercise self-efficacy can help individuals understand and utilize social support more deeply, thereby influencing their fitness behavior (Luo et al., 2024).

The emotion regulation model proposed by Dodge and Garber (1991) and other researchers points out that psychological resilience plays a significant internal and external role in emotion regulation. Specifically, individuals with high resilience tend to have a strong sense of self-efficacy, that is, full of confidence in their ability to complete specific tasks. When they encounter challenges and pressures in the process of fitness, they can adjust their emotions more effectively. This emotion regulation ability helps to maintain a positive emotional state, thus enhancing the intrinsic motivation and sustainability of fitness behavior, thereby positively impacting their self-directed fitness behavior. In addition, exercise self-efficacy can not only adjust psychological resilience, but also help individuals make more efficient use of social support resources, so as to further promote the enhancement of autonomous fitness behavior (Schäfer et al., 2024). This mechanism can be explained by the regulatory flexibility theory of Bonanno (2005). The theory emphasizes that the ability of individuals to adjust emotional experience and coping strategies mainly depends on the demands and feedback of the situation. In the context of fitness, psychological resilience may play a regulatory role, affecting how individuals combine social support and self-efficacy to adapt to various changing situations in the process of fitness, and then affect their fitness behavior (Bonanno, 2005).

The comprehensive analysis indicates that exercise self-efficacy plays an important role in the regulation of psychological resilience on perceived social support. Improving the exercise self-efficacy of individuals can not only help to enhance their psychological resilience, but also promote them to obtain and use social support resources more effectively, thereby positively impacting their self-directed fitness behavior. These research findings offer a solid theoretical foundation and practical guidance for enhancing college students’ mental health and fitness behaviors. Thus, the hypothesis H3, which posits that psychological resilience is moderated by exercise self-efficacy through the mediating mechanism of perceived social support, has been effectively validated.

## Conclusion

(1) Psychological resilience can directly promote college students’ autonomous fitness behavior, and can promote their autonomous fitness behavior through perceived social support. Perceived social support plays a partial mediating role between psychological resilience and autonomous fitness behavior, and this mediating effect can be moderated by exercise self-efficacy.

(2) Compared with high exercise self-efficacy, for college students with low exercise self-efficacy, perceived social support plays a stronger mediating role.

### Limitations and future directions

This study provides valuable insights into the interrelationships among psychological resilience, perceived social support, and fitness behaviors in college students. However, several limitations warrant consideration. First, the cross-sectional design precludes establishing causal relationships between the observed associations of psychological resilience, perceived social support, and fitness behaviors. Second, reliance on self-reported measures introduces potential recall bias and social desirability bias, compromising both data accuracy and contextual specificity. Third, the sample was drawn from a limited number of geographic regions, which may limit the generalizability of the findings to other populations. Also, while this study controlled for gender as a covariate, it did not systematically conduct gender-specific analyses—a methodological limitation that may have obscured critical mechanisms underlying gender differences. However, our current analytical framework lacked the capacity to rigorously explore this dimension. Additionally, the study did not control for potential confounding variables, such as socioeconomic status or pre-existing fitness habits, which could influence the observed relationships.

Despite these limitations, the findings underscore the critical need for multi-tiered strategies to promote college students self-directed exercise engagement. This investigation advances existing scholarship by systematically analyzing how psychological resilience interacts with self-regulated exercise patterns in collegiate populations. A novel contribution of this research lies in positioning exercise self-efficacy as a moderating variable within the theoretical framework. Earlier studies have predominantly conceptualized self-efficacy as an independent predictor, overlooking its synergistic relationships with resilience mechanisms and social support systems. By modeling exercise self-efficacy as a moderating mechanism, this study elucidates the dynamic interplay through which these psychological factors jointly shape exercise adherence.

To address the limitations and build on the current findings, future research should prioritize longitudinal mixed methods designs. Such approaches can provide a more comprehensive understanding of the dynamic interplay between psychological resilience, social support, and fitness behaviors over time. Moreover, future studies should consider incorporating objective measures of fitness behaviors (e.g., wearable devices) to enhance data accuracy and reduce reliance on self-reports. Expanding the sampling to include broader geographic and demographic diversity would improve the findings generalizability. Finally, Future investigations should prioritize examining gender-cultural context interactions.

## Data Availability

The original contributions presented in the study are included in the article/Supplementary material, further inquiries can be directed to the corresponding authors.
